# Impact of air pollution exposure during various periods of pregnancy on term birth weight: a large-sample, retrospective population-based cohort study

**DOI:** 10.1007/s11356-020-10705-3

**Published:** 2020-09-11

**Authors:** Li Shang, Liyan Huang, Liren Yang, Longtao Leng, Cuifang Qi, Guilan Xie, Ruiqi Wang, Leqian Guo, Wenfang Yang, Mei Chun Chung

**Affiliations:** 1 Department of Obstetrics and Gynecology, Maternal & Child Health Center, The First Affiliated Hospital of Xi’an Jiaotong University, Xi’an, Shaanxi People’s Republic of China; 2 School of Public Health, Xi’an Jiaotong University Health Science Center, Xi’an, Shaanxi People’s Republic of China; 3 School of Computer Science & Engineering, University of Electronic Science and Technology of China, Chengdu, Sichuan People’s Republic of China; 4 Department of Public Health and Community Medicine, Tufts University School of Medicine, Boston, Massachusetts USA

**Keywords:** Maternal exposure, Air pollution, Air quality index, Term birth weight, Term low birth weight, Macrosomia

## Abstract

Previous studies have suggested that maternal exposure to air pollution might affect term birth weight. However, the conclusions are controversial. Birth data of all term newborns born in Xi’an city of Shaanxi, China, from 2015 to 2018 and whose mother lived in Xi’an during pregnancy were selected form the Birth Registry Database. And the daily air quality data of Xi’an city was collected from Chinese Air Quality Online Monitoring and Analysis Platform. Generalized additive models (GAM) and 2-level binary logistic regression models were used to estimate the effects of air pollution exposure on term birth weight, the risk term low birth weight (TLBW), and macrosomia. Finally, 321521 term newborns were selected, including 4369(1.36%) TLBW infants and 24,960 (7.76%) macrosomia. The average pollution levels of PM_2.5_, PM_10_, and NO_2_ in Xi’an city from 2015 to 2018 were higher than national limits. During the whole pregnancy, maternal exposure to PM_2.5_, PM_10_, SO_2_, and CO all significantly reduced the term birth weight and increased the risk of TLBW. However, NO_2_ and O_3_ exposure have significantly increased the term birth weight, and O_3_ even increased the risk of macrosomia significantly. Those effects were also observed in the first and second trimesters of pregnancy. But during the third trimester, high level of air quality index (AQI) and maternal exposure to PM_2.5_, PM_10_, SO_2_, NO_2_, and CO increased the term birth weight and the risk of macrosomia, while O_3_ exposure was contrary to this effect. The findings suggested that prenatal exposure to air pollution might cause adverse impacts on term birth weight, and the effects varied with trimesters and pollutants, which provides further pieces of evidence for the adverse effects of air pollution exposure in heavy polluted-area on term birth weight.

**Electronic supplementary material:**

The online version of this article (10.1007/s11356-020-10705-3) contains supplementary material, which is available to authorized users.

## Introduction

With the rapid development of global industrialization and urbanization, the problem of ambient air pollution has been getting increasingly prominent, which is the largest health environmental risk affecting all regions, socioeconomic groups, and age groups (Brauer et al. 2016). Some studies have suggested that air pollutants might increase the risk of cardiovascular and respiratory diseases through inducing some abnormal reactions, such as oxidative stress and DNA methylation (Chen et al. 2020a; Chu et al. 2015; Kaufman et al. 2016).

As the most sensitive populations, pregnant women and newborns are more vulnerable to the adverse effects of ambient air pollution. Some epidemiological studies indicated that maternal exposure to air pollution had adverse effects on infant health, such as adverse birth outcome and respiratory and neurodevelopmental effects (Backes et al. 2013; Forns et al. 2018; Korten et al. 2017; Pedersen et al. 2017; Shang et al. 2019; Yorifuji et al. 2015). Among them, birth weight is one of the most important predictors of morbidity and mortality in neonates and childhood and adult morbidity. Abnormal birth weight (including low birth weight (LBW) and macrosomia) is generally associated with several long-term chronic diseases in adults, including cardiovascular disease, type 2 diabetes, and certain cancers (Moraitis et al. 2014; Zhang et al. 2014).

Increasing evidence suggested that ambient air pollution exposure might affect fetal growth and result in abnormal birth weight. A retrospective cohort study based on a large sample suggested that PM_2.5_ exposure during pregnancy might increase the risk of term low birth weight (TLBW), but not in other air pollutants, and it also suggested that 3% of LBW cases can be directly attributed to residential PM_2.5_ exposure higher than 13.8 μg/m^3^ during pregnancy (Smith et al. 2017). And this conclusion was consistent with other similar studies (Janssen et al. 2017; Li et al. 2019). But He et al. found that with the increase of SO_2_ during pregnancy, birth weight was significantly decreased, while birth weights were significantly increased with NO_2_ exposure (He et al. 2018). Therefore, studies on the effect of maternal exposure to air pollution on birth weight are still not uniform. And most studies have only estimated the effect of air pollution exposure during the whole pregnancy and lacked of exposure assessment at different trimesters of pregnancy, at which fetus will have different growth and development mechanisms that might lead to inconsistent effects of air pollution. In addition, few studies evaluated the effects of air pollution during pregnancy on birth weight gain and the risk of macrosomia.

Therefore, we conducted a retrospective cohort study with large sample in Xi’an city of Shaanxi province in northwestern China. In this study, the birth data of term newborns born from 2015 to 2018 in Xi’an city was collected from the birth registration system, and their birth weight was recorded. And prenatal exposure levels of air pollution at each trimester of pregnancy were calculated to estimate the effects of air pollution exposure during various trimesters of pregnancy on term birth weight, the risk of TLBW, and macrosomia.

## Methods

### Study population

**Fig. 1:**
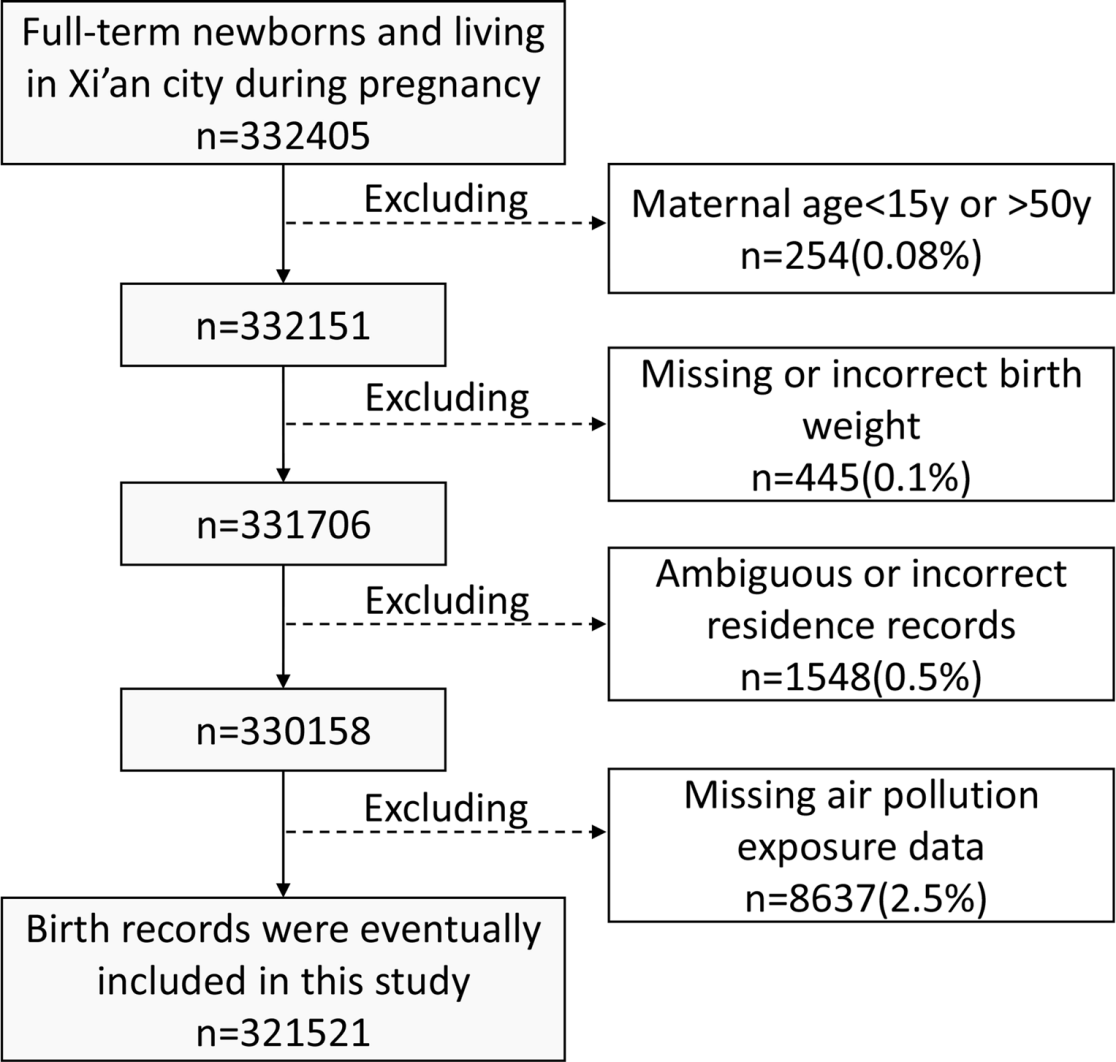
Flow diagram outlining the selection of study population

This study including all births in Xi’an city of Shaanxi province in China from January 2015 to December 2018 (*n* = 536993). The data on newborns were collected from the Birth Registry Database covering all midwifery clinics and hospitals in Xi’an city (Fig. 1).

In a total of 536993 newborns, we only selected newborns who lived in Xi’an city during pregnancy and recorded the exact residential address to the street as study objects (*n* = 349069). Among them, only term newborns with a gestational age of ≥ 37 weeks and < 42 weeks were enrolled (*n* = 332405). And those records in which maternal gestational age was missing or less than 15 years or older than 50 years were excluded (*n* = 254). In addition, some records with missed information of birth weight (*n* = 445) and ambiguous or incorrect residence (*n* = 1548) were also excluded. In addition, since exposure data on air pollution prior to May 13, 2014, were not available, we excluded pregnant women who were pregnant before this date (*n* = 8637). Finally, 321,521 newborns’ records were included in our study. This study was approved by the Medical Ethics Committee of the First Affiliated Hospital of Xi’an Jiaotong University (No. XJTU1AF2017LSK-106).

### Air pollution exposure

Real-time data of ambient air pollution exposure from May 13, 2014, to December 30, 2018, were provided by Chinese Air Quality Online Monitoring and Analysis Platform (https://www.aqistudy.cn/). It covered 13 air pollution monitoring sites in Xi’an city of Shaanxi province and originated mainly from the real-time data recorded by the Ministry of Ecology and Environment of the People’s Republic of China (http://www.mee.gov.cn/). Differential optical absorption spectroscopy method was used to analyze the concentration of NO_2_, SO_2_, CO, and O_3_, and the tapered element oscillating microbalance method was used to analyze the concentration of PM_2.5_ and PM_10_ automatically. AQI is an important indicator that presents the overall air pollution level, which is calculated according to the new ambient air quality standards (GB3095-2012). The definition of the used AQI is presented in the Supplementary Material (Bao et al. 2015), and the higher AQI presents more serious air pollution. The proportion of missing values is 0.5% for air pollutants. The past and future 7-day averages were used to impute the missing daily exposure data.

Maternal exposure estimates were calculated by pollutant concentrations measured at the nearest monitoring station to their residence. Prior to the assessment, the 24-h average of AQI, PM_2.5_, PM_10_, SO_2_, NO_2_, and CO and the 8-h average of O_3_ over all monitors were calculated, and the residences of the pregnant woman during pregnancy were collected from the birth registration information. Then, the latitudes and longitudes of all monitors and maternal residences were converted by geocoding. After the latitude and longitude distance differences from the residences to the monitoring stations were calculated, the ambient air pollution concentrations of the nearest monitor were assigned as individual exposure estimates. Finally, based on the start and end dates of each trimester of pregnancy, we calculated the average exposure levels of air pollution for each pregnant woman during the whole pregnancy (from pregnancy to delivery), the first trimester (from pregnancy to the 13th week of gestation), the second trimester (from the 14th to 27th week of gestation), and the third trimester (from the 28th week of gestation to delivery).

### Outcomes and covariate data

Our main outcomes were term birth weight, TLBW, and macrosomia. Term birth weights for each newborn were collected from birth records directly. Term low birth weight (TLBW) was defined as birth weight less than 2500 g and gestational age of 37 weeks or more. And macrosomia was defined as birth weight equal or more than 4000 g. Meanwhile, maternal age, gestational age, ethnicity, and the date of birth of each newborn were all recorded. Pregnancy date was calculated based on birth date and gestational age to classify the season of beginning pregnancy (including warm season (from April to September) and cold season (from October to March of the next year)). And average temperature exposures during pregnancy were calculated based on the date of birth, gestational age, and hourly temperature distribution in Xi’an city from 2015 to 2018.

### Statistical analysis

The average exposure values of AQI, PM_2.5_, PM_10_, SO_2_, NO_2_ CO, and O_3_ from 2015 to 2018 in Xi’an city were estimated based on hourly average exposure levels by the weighted average method. Generalized additive model (GAM) fitting in the Gaussian distribution was performed to evaluate the nonlinear relationship of air pollution exposure and birth weight (Shang et al. 2019). After fitting the related confounders, including maternal age, gestational age ethnicity, and pregnancy season, the degrees of freedom of confounders were calculated by using the Akaike information criterion (AIC). Then, the basic model was finally established as follows:

$$ \mathrm{Log}\ \left[E\left({Y}_t\right)\right]={\upbeta \mathrm{X}}_{\mathrm{t}}+ ns\ \left(\mathrm{maternal}\ \mathrm{age}, df=4\right)+ ns\ \left(\mathrm{gestational}\ \mathrm{age}, df=24\right)+\mathrm{as}.\mathrm{factor}\ \left(\mathrm{ethnicity}\right)+\mathrm{as}.\mathrm{factor}\ \left(\mathrm{season}\right)+\upalpha $$

*E*(*Y*_*t*_) is the expected birth weight for newborn *t*, *X*_*t*_ represents the exposure level of air pollution for pregnant women *t*. Based on this model, the effects of per 10 unit increase of AQI, per 10 μg/m^3^ increase of PM_2.5_, PM_10_, SO_2_, NO_2_, O_3_, and per 0.1 mg/m^3^ increase of CO on term birth weight, were evaluated.

In addition, macrosomia or low birth weight infants were selected as the case group, while those infants with normal birth weight (2500 g ≤ birth weight and < 4000 g, *n* = 292192) were selected as a reference, and then 2-level binary logistic regression model was performed to investigate the associations between air pollution exposure and the risk of TLBW and macrosomia. The odds ratios (*OR*s) and 95% confidence intervals (*CI*s) were calculated for the effects of per 0.1 mg/m^3^ increase of CO and per 10 unit increase of AQI, PM_2.5_, PM_10_, NO_2_, SO_2_, and O_3_ on the risk of TLBW and macrosomia.

Due to the non-significant relationship between average temperature and birth weight, we only included maternal age, gestational age, ethnic group, and pregnancy season as confounding factors in the final GAM and binary logistic regression model. All analyses were conducted using “mgcv (Mixed GAM Computation Vehicle)” and “glm” package and in R version 3.5.3. Significant results were considered at a *P* value lower than 0.05 (*P* < 0.05).

## Results

### Basic characteristic

A total of 321,521 newborns’ birth records were included in this study. The mean (10th–90th percentile) maternal age was 28.48 (23–34) years. And the Han ethnic group accounted for the majority (96.19%). The mean gestational age of newborns was 275.65 (265–287) days. And the mean birth weight was 3328.74 g. Among them, the incidence of TLBW and macrosomia were 1.36% (4369 cases) and 7.76% (24960 cases), respectively (Table 1).

**Table 1:** Basic characteristics

Variable	*N* (%) or mean ± SD	Mean birth weight	TLBW [*n* (%)]	Macrosomia [*n* (%)]
Total population	321521	3328.74	4369 (1.36)	24,960 (7.76)
Gestational age (days)	275.65 ± 7.46	-	-	-
Maternal age (years)	28.48 ± 4.34	-	-	-
Maternal age (years)				
<25	55,646 (17.31)	3285.12	937 (1.68)	3401 (6.11)
25-29	149,636 (46.54)	3332.51	4618 (3.09)	11,351 (7.59)
30-34	86,586 (26.93)	3347.63	1868 (2.16)	7442 (8.59)
35-39	25,606 (7.96)	3340.55	363 (1.42)	2375 (9.28)
≥40	4047 (1.26)	3301.66	70 (1.73)	391 (9.66)
Ethnicity				
Han	318,365 (99.01)	3328.86	4334(1.36)	24678(7.75)
Others	3156 (0.99)	3317.22	35(1.11)	282(8.94)
Birth season				
Warm	160,680 (49.98)	3356.95	2247 (1.40)	12,339 (7.68)
Cold	160,841 (50.02)	3366.89	2122 (1.32)	12,621 (7.85)

### The distribution of air pollution in Xi’an, Shaanxi province, from 2015 to 2018

The average levels of PM_2.5_, PM_10_, and NO_2_ in Xi’an city of Shaanxi province from 2015 to 2018 were all higher than the National Ambient Air Quality Standard (GB 3095–2012) limits. Among them, the concentration of PM_10_ exposure was the most severe. In addition, the exposure levels of SO_2_, CO, and O_3_ were lower than the National Ambient Air Quality Standard (GB 3095–2012) limits (Table 2).

**Table 2:** The exposure levels of air pollution in Xi’an, Shaanxi province, China

	Mean ± SD	*P* (25)	Median	*P* (75)	Concentration limits*
AQI	102.70 ± 60.50	66.18	88.31	116.40	-
PM_2.5_ (μg/m^3^)	64.28 ± 51.08	33.43	47.79	74.68	35
PM_10_ (μg/m^3^)	126.11 ± 77.96	73.85	103.58	155.31	70
SO_2_ (μg/m^3^)	21.09 ± 14.53	11.13	16.51	27.01	60
NO_2_ (μg/m^3^)	49.75 ± 18.89	35.60	45.96	60.73	40
CO (mg/m^3^)	1.53 ± 0.68	1.03	1.33	1.78	4
O_3_ (μg/m^3^)	48.56 ± 29.00	23.71	43.58	68.31	160

*Concentration limits of PM_2.5_ and PM_10_, SO_2_ and NO_2_: the maximum allowable value of the average concentration within a year; Concentration limit of CO: the maximum allowable value of the average concentration within any 24 h; Concentration limit of O_3_: the maximum allowable value of the average concentration within any 8 h

### The effect of maternal exposure to air pollution during whole pregnancy on term birth weight

During the whole pregnancy, per 10 μg/m^3^ increase of PM_2.5_, PM_10_, SO_2_, and per 0.1 mg/m^3^ increase of CO exposure all significantly reduced the term birth weight of newborns (*β*(95%*CI*) values were − 2.739 (− 3.693, − 1.785), − 2.458 (− 3.116, − 1.800), − 3.982 (− 5.511, − 2.453) and − 1.511 (− 1.970, − 1.053), respectively) and increased the risk of TLBW (*OR* (95%*CI*) values were 1.025 (1.005–1.045), 1.035 (1.020–1.049), 1.034 (1.004–1.065), and 1.013 (1.004–1.023), respectively). However, every 10 μg/m^3^ increase in NO_2_ (1.734 (0.533, 2.935)) and O_3_ (4.531 (3.239, 5.823)) significantly increased the birth weight of term newborns, and O_3_ even increased the risk of macrosomia significantly (1.028 (1.015–1.040)) (Table 3). In the generalized additive model, the exposure levels of air pollutants during the whole pregnancy explained 11.3 to 11.7% of the deviance in birth weight change.

**Table 3:** The effect of maternal exposure to air pollution during whole pregnancy on term birth weight

Air pollutants	Birth weight			TLBW			Macrosomia		
	*β*	95%CI	*P*	OR	95%CI	*P*	OR	95%CI	*P*
AQI	− 2.853	− 3.743, − 1.963	< 0.001	1.029	1.010–1.048	0.003	0.984	0.976–0.993	< 0.001
PM_2.5_	− 2.739	− 3.693, − 1.785	< 0.001	1.025	1.005–1.045	0.018	0.986	0.977–0.995	0.003
PM_10_	− 2.458	*−3.116, − 1.800*	< 0.001	1.035	1.020–1.049	<0.001	0.989	0.983–0.996	0.001
SO_2_	*− 3.982*	− 5.511, − 2.453	< 0.001	1.034	1.004–1.065	0.032	0.978	0.963–0.993	0.004
NO_2_	1.734	0.533, 2.935	0.005	0.991	0.964–1.017	0.476	0.988	0.977–1.000	0.044
CO	− 1.511	− 1.970, − 1.053	< 0.001	1.013	1.004–1.023	0.008	0.992	0.987–0.996	< 0.001
O_3_	4.531	3.239, 5.823	< 0.001	0.952	0.925–0.980	< 0.001	1.028	1.015–1.040	< 0.001

*Note*: Above models were all adjusted for maternal age, gestational age, ethnicity, and pregnancy season. Infants with normal term birth weight (≤ 2500 g birth weight and < 4000 g, *n* = 292192) were used as the control group while assessing the impact of air pollution exposure on the risk of TLBW and macrosomia. AQI means air quality index, which is an important indicator that presents overall air pollution level

### The effect of maternal exposure to air pollution during various periods of pregnancy on term birth weight, TLBW, and macrosomia

**Fig. 2:**
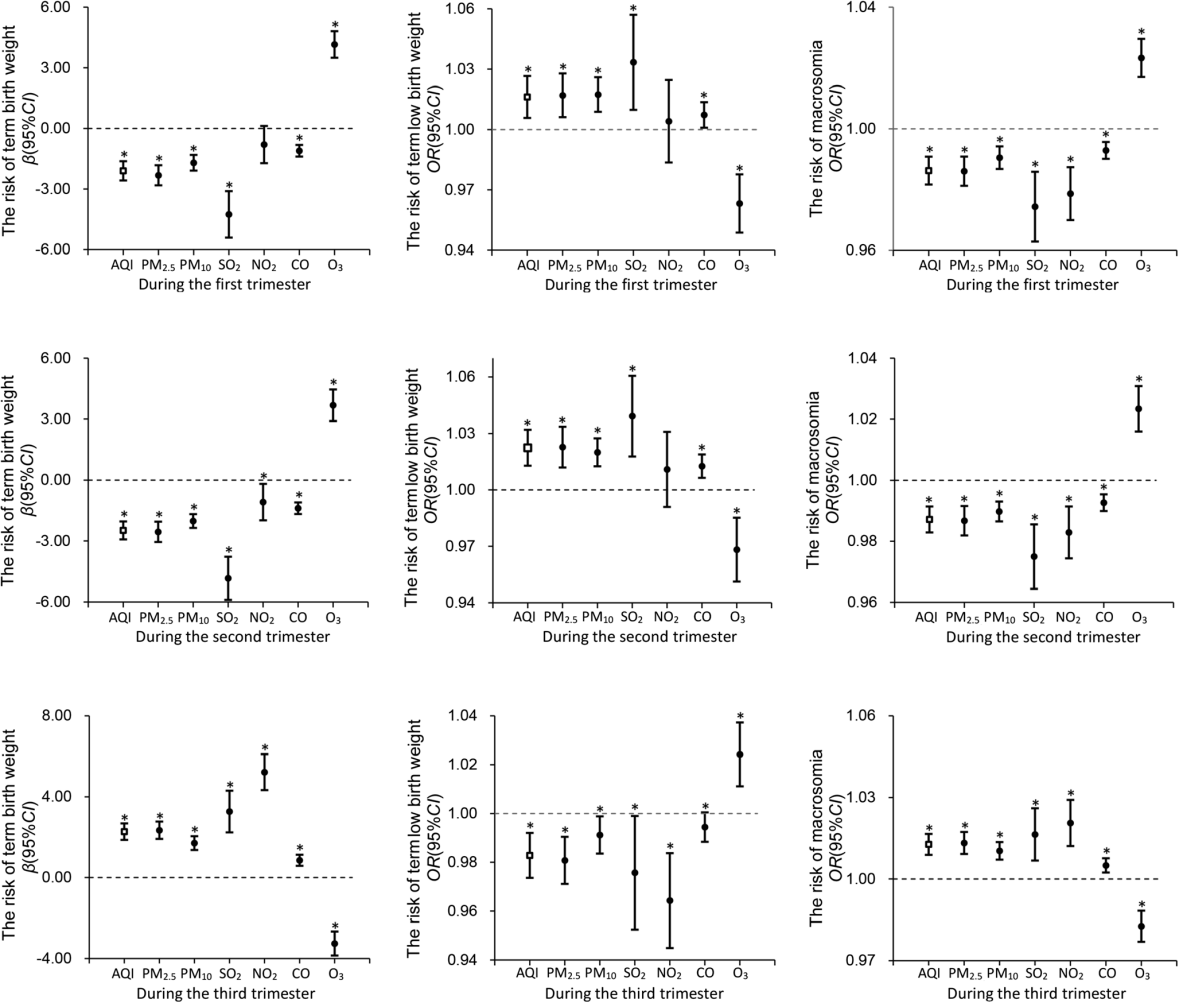
The effect of maternal exposure to air pollution during various periods of pregnancy on term birth weight, TLBW, and macrosomia. *Note*: The above models were all adjusted for maternal age, gestational age, and ethnicity. Infants with normal term birth weight (≤ 2500 g birth weight and < 4000 g, *n* = 292192) were used as the control group while assessing the impact of air pollution exposure on the risk of TLBW and macrosomia. AQI means air quality index, which is an important indicator that presents the overall air pollution level. And * means *P* < 0.05

The effect of air pollution exposure during the first trimester on term birth weight was consistent with that in the whole pregnancy. It showed that high levels of AQI and maternal exposure to PM_2.5_, PM_10_, SO_2_, and CO during the first trimester were negatively associated with term birth weight (AQI: − 2.100 (− 2.576, − 1.624); PM_2.5_: − 1.700 (− 2.088, − 1.312); PM_10_: − 4.258 (− 5.408, − 3.108); − 4.258 (− 5.408, − 3.108); CO: − 1.105 (− 1.394, − 0.816)) and positively associated with the incidence of TLBW (AQI: 1.016 (1.006–1.027); PM_2.5_: 1.017 (1.006–1.028); PM_10_: 1.017 (1.009–1.026); SO_2_: 1.033 (1.010–1.057); CO: 1.007 (1.001–1.014)). But maternal O_3_ exposure increased the risk of term birth weight (*β* = 4.150, 95%*CI*: 3.493–4.807) and macrosomia (*OR* = 1.023, 95%*CI*: 1.017–1.030). No significant association was found between maternal NO_2_ exposure and birth weight during early pregnancy (Fig. 2, Table S2).

The effect of maternal exposure to air pollution during the second trimester on term birth weight was also consistent with that in the whole pregnancy. It showed that a high level of AQI and maternal exposure to PM_2.5_, PM_10_, SO_2_, and CO during the first trimester reduced the term birth weight and increased the risk of TLBW. But O_3_ exposure increased the risk of term birth weight and macrosomia (Fig. 2, Table S2).

Particularly, the effect of air pollution exposure during the third trimester on birth weight was opposite to that in the first or second trimester. High levels of AQI and maternal exposure to PM_2.5_, PM_10_, SO_2_, NO_2_, and CO during the third trimester have significantly increased newborn’s birth weight (AQI: 2.274 (1.865, 2.683); PM_2.5_: 2.344 (1.914, 2.774); PM_10_: 1.705 (1.363, 2.047); SO_2_: 3.270 (2.237, 4.303); NO_2_: 5.219 (4.331, 6.107); CO: 0.855 (0.580, 1.130)). Conversely, O_3_ exposure reduced the term birth weight (*β* = − 3.258, 95%*CI*: - 3.852, - 2.664) and increases the risk of TLBW (*OR* = 1.024, 95%*CI*: 1.011–1.037) significantly for newborns (Fig. 2, Table S2). In the generalized additive model, the exposure levels of air pollutants during each trimester of pregnancy explained 11.3 to 11.9% of the deviance in birth weight changes.

## Discussion

### Main results

Based on a large sample study, we found that maternal exposure to air pollution might cause significant impacts on birth weight. During the entire pregnancy, as well as the first and second trimesters of pregnancy, maternal exposure to PM_2.5_, PM_10_, SO_2_, and CO significantly reduced the term birth weight of newborns and increased the risk of TLBW, while O_3_ exposure increased term birth weight and the risk of macrosomia. But during the third trimester, AQI, PM_2.5_, PM_10_, SO_2_, NO_2_, and CO exposure increased the term birth weight and the risk of macrosomia, while O_3_ exposure had the opposite effect.

### The effect of air pollution exposure on birth weight

#### Air pollution exposure during the whole pregnancy and the first and second trimester

Our study found that a high level of AQI and maternal exposure to PM_2.5_, PM_10_, SO_2_, and CO during the whole pregnancy and the first and second trimesters significantly reduced birth weight and increased the risk of TLBW. This conclusion had also been observed in other similar studies (Arroyo et al. 2019; Chen et al. 2018; He et al. 2018; Li et al. 2019; Smith et al. 2017; Yorifuji et al. 2015). A retrospective cohort study of 540,365 singleton term live births conducted in London suggested that PM_2.5_ exposure throughout pregnancy was correlated with increased risk of long-term LBW (Smith et al. 2017). Another study, conducted in Guangdong province of China, also showed an increased risk of LBW associated with PM_2.5_, PM_10_, and NO_2_ in the first trimester (Liu et al. 2019). In addition, similar conclusions have also been found in some meta-analysis and reviews (Guo et al. 2019; Li et al. 2017). And a meta-analysis also suggested that pregnant women who smoked were underweight, overweight/obese, or had lower socioeconomic status had an increased risk of having a child with LBW when exposed to ambient PM_2.5_, SO_2_, and NO_2_ (Westergaard et al. 2017). However, no significant association appeared in studies by Laurent et al. (2013) and Lavigne et al. (2016b) for air pollution exposure, which may be because the concentrations of air pollution in those study areas were relatively cleaner, so that the adverse effects did not show up. However, contrary to our findings, most studies indicated that maternal exposure to O_3_ might reduce birth weight or increase the risk of LBW (Brauer et al. 2008; Laurent et al. 2013; Li et al. 2017). And nearly none of the studies has found the effect of O_3_ exposure on birth weight increase. Only one study reported that O_3_ exposure in the second trimester reduced the risk of low birth weight (Ebisu and Bell 2012), which may indicate its possible increased effect on birth weight. Based on all the results of these studies, we did not find a consistent conclusion of dose-response pattern among studies. In other words, the association between air pollution and term birth weight is still controversial.

#### Air pollution exposure during the third trimester

Conversely, we found that maternal exposure to air pollutants other than O_3_ during the third trimester might increase birth weight and the risk of macrosomia. Only few studies drew consistent conclusions with our study (Chen et al. 2020b; Li et al. 2019; Zhao et al. 2018). A nationwide prospective cohort study in China has suggested that per 10 μg/m^3^ increase of PM_2.5_ concentration over the third trimesters obviously increased the risk of macrosomia (*OR*: 1.033; 95%*CI*: 1.026–1.039) (Chen et al. 2020b). In addition, Li et al. found that exposure to NO_2_ in the third trimester significantly increased birth weight, while O_3_ exposure decreased it, which was consistent with our conclusion (Li et al. 2019). However, contrary to our findings, most previous related studies still suggested that prenatal exposure to PM_2.5_, PM_10_, and CO in the third trimester might cause birth weight reduction significantly (Chen et al. 2018; Guo et al. 2017; He et al. 2018; Santos Vde et al. 2016; Ye et al. 2018). Several reasons, such as the concentration difference and misclassification of air pollution, and co-linearity questions might explain the inconsistent results among these studies. It suggests that the effects of air pollution exposure in the third trimester on birth weight, especially macrosomia, remain to be determined further.

### Potential mechanism

The mechanism by which maternal air pollution exposure affects birth weight is still unclear, but some population studies have supported that some abnormal reactions, such as the gene methylation level in cord blood (He et al. 2018) and mitochondrial DNA (mtDNA) content in placenta (Clemente et al. 2016), might mediate the effect of air pollution exposure on birth weight reduction. Based on previous pieces of evidence, we concluded that air pollution enters the human body through the respiratory tract, causing some abnormal reactions such as oxidative stress (Nagiah et al. 2015), inflammatory response (Pope et al. 2016), and DNA methylation (Plusquin et al. 2017). Mitochondria, the organelles that regulate energy production, lack protection and repair mechanisms, which are easily damaged by reactive oxygen species produced by oxidative stress (Janssen et al. 2012). Such damage might eventually lead to the reduced numbers of mitochondria and the damage of energy flow, thus resulting in the reduction of material energy supply and even the reduction of birth weight (van den Hooven et al. 2012). In addition, it is reported that air pollutants also caused histopathological changes and vascularization injuries of the placenta, which might further lead to nutrient and waste transfer obstruction and abnormal cellular growth, thus reducing the birth weight of newborns (Yue et al. 2019).

On the contrary, we also found that some air pollutants at certain periods increased term birth weight, which might be related to the increase of leptin and adiponectin in cord blood. Several studies have reported that prenatal PM_2.5_ and traffic-related air pollution exposure increased the levels of umbilical blood leptin and high-molecular-weight adiponectin in cord blood (Alderete et al. 2018; Bass et al. 2013; Lavigne et al. 2016a), which were found to be positively associated with increased birth weight (Mantzoros et al. 2009; Tsai et al. 2004). And the levels of leptin in the third trimester are higher (Stefaniak et al. 2019), which might cause its level to be more sensitive to air pollution exposure in the third trimester. Therefore, it provided some molecular basis for the effect of air pollution exposure in the third trimester on birth weight increase. And air pollution exposure in the third trimester also might contribute to neonatal weight gain by mediating the high risk of gestational diabetes mellitus (Kc et al. 2015). In addition, the hypothesis of “thrifty phenotype” also can explain our findings that the effect of air pollution exposure in the first and second trimester is opposite to that in the third trimester to some extent (Hales and Barker 2001). However, further researches are still needed to confirm the above hypothesis and clarify the mechanism of air pollution on birth weight.

### Advantage and limitation

Our study had some advantages compared with other studies. Firstly, it is a large sample study based on 321,521 pregnant women and their infants, and very strict inclusion and exclusion criteria were adopted, which helps to control information bias. Secondly, our research was based on the birth registration data of Xi’an city in China from 2015 to 2018, and the exposure level of air pollution was very high and the exposure range is wide. Therefore, compared with other studies conducted in cleaner areas, our research can better reflect the adverse effects of high exposure levels of air pollution on birth weight. Thirdly, through the individual exposure assessment based on exact residence (down to street) during pregnancy, we comprehensively estimated the impact of air pollution exposure during four different periods of pregnancy on the term birth weight and the risk of TLBW and macrosomia and drawn comprehensive and significant conclusions.

But several limitations should be considered in interpreting the results of this study. Due to the limited information collected by the birth registration system, some confounding factors were not considered in our study, such as diet, maternal BMI, sex of newborns, and the history of diseases during pregnancy. However, previous studies have found the effect value changed little whether or not the above risk factors were adjusted (Brauer et al. 2008; Kashima et al. 2011; Kim et al. 2007). And we excluded premature infants most of them accompanied by disease history during pregnancy, and only estimated the effect for air pollution exposure on term infants, so as to control the bias caused by disease to a certain extent. In addition, exposure misclassification might exist due to the lack of information on maternal activity and residential mobility during pregnancy. However, exposure misclassification is more likely to be non-differential (Wang et al. 2018).

## Conclusion

Our research has suggested that the effects of air pollution on birth weight varied with exposure periods and pollutants. During the entire pregnancy, as well as the first and second trimesters of pregnancy, maternal exposure to high AQI, PM_2.5_, PM_10_, SO_2_, and CO significantly reduced the term birth weight of newborn and increased the risk of TLBW, while O_3_ exposure increased term birth weight and the risk of macrosomia. However, during the third trimester, AQI, PM_2.5_, PM_10_, SO_2_, NO_2_, and CO exposure increased the term birth weight and the risk of macrosomia, while O_3_ exposure had the opposite effect.

## Electronic supplementary material

ESM 1 (DOCX 24 kb)

## Data Availability

The datasets generated and/or analyzed during the current study are not publicly available since we are still conducting other major analyses based on this database, but they are available from the corresponding author on reasonable request.
