# Supplementary material for: Impact of air pollution exposure during various periods of pregnancy on term birth weight: a large-sample, retrospective population-based cohort study
Source: Environ Sci Pollut Res Int. 2020 Sep 11;28(3):3296–306. doi: 10.1007/s11356-020-10705-3 (PMC7788013; doi:10.1007/s11356-020-10705-3)
Supplement: Supplementary file 1 — (DOCX 24 kb) [file 11356_2020_10705_MOESM1_ESM.docx]

**Supplementary Material**

**Impact of air pollution exposure during various periods of pregnancy on term birth weight: a large-sample, retrospective population-based study**

Li Shang^a,b,1^, Liyan Huang^a,b,1^, Liren Yang^a,b^, Longtao Leng^c^, Cuifang Qi^a^, Guilan Xie^a,b^, Ruiqi Wang^a,b^, Leqian Guo^a^, Wenfang Yang^a^ *, Mei Chun Chung^d^

**^a^** Department of Obstetrics and Gynecology, Maternal & Child Health Center, The First Affiliated Hospital of Xi’an Jiaotong University, Xi’an, Shaanxi, P.R. China

^b^ School of Public Health, Xi’an Jiaotong University Health Science Center, Xi’an, Shaanxi, P.R. China

^c^ School of Computer Science & Engineering, University of Electronic Science and Technology of China, Chengdu, Sichuan, P.R. China

^d^ Department of Public Health and Community Medicine, Tufts University School of Medicine, Massachusetts Boston, United States of America

**^1^ These authors contributed equally to the work.**

***Corresponding author:** Wenfang Yang, Ph.D, Department of Obstetrics and Gynecology, Maternal & Child Health Center, the First Affiliated Hospital of Xi’an Jiaotong University No. 277, West Yanta Road. Xi'an, Shaanxi Province 710061, P.R.China

**Supplementary formulae:**

**The definition of AQI**

For one pollutant i (PM_2.5_, PM_10_, CO, SO_2_, NO_2_ and O_3_), the air quality index (AQI) is calculated by

$${AQI}_{i}=\frac{{IAQI}_{Hi}-{IAQI}_{Lo}}{C_{Hi}-C_{Lo}}\left( C_{i}-C_{Lo} \right)+{IAQI}_{Lo}$$

$$AQI=max({AQI}_{i})$$

where AQI*_i_* is the index for the pollutant *i* (*i.e.*, SO_2_, NO_2_, CO, O_3_, PM_2.5_ and PM_10_); C*_i_* is the monitored ambient concentration of pollutant *i*; C*_Hi_* and C*_Lo_* are the nearly upper and lower breakpoints corresponding to C*_i_*, respectively; IAQI is the individual air quality index, and IAQI*_Hi_* and IAQI*_Lo_* are the sub-indices corresponding to C*_Hi_* and C*_Lo_*, respectively. The overall AQI is the maximum sub-AQI of all pollutants.

**Supplementary tables:**

**Table S1** Air pollution exposure levels at various period of pregnancy

| Variable | Whole pregnancy | |  | | First trimester | |  | | Second trimester | |  | | Third trimester | |
| --- | --- | --- | --- | --- | --- | --- | --- | --- | --- | --- | --- | --- | --- | --- |
|  | Mean ± SD | Rang | | Mean ± SD | | Rang | | Mean ± SD | | Rang | | Mean ± SD | | Rang |
| AQI | 104.34±16.76 | 42.39-152.75 | | 105.26±36.09 | | 31.92-235.21 | | 103.78±37.22 | | 31.92-235.21 | | 104.18±41.83 | | 28.51-259.20 |
| PM_2.5_ (μg/m^3^) | 66.09±15.71 | 11.46-114.81 | | 66.75±32.61 | | 6.15-191.15 | | 65.60±33.62 | | 6.15-191.15 | | 65.07±36.95 | | 5.38- 221.80 |
| PM_10_ (μg/m^3^) | 128.10±22.34 | 40.61-182.12 | | 130.42±45.79 | | 23.02-271.60 | | 127.38±47.61 | | 23.02-271.60 | | 126.47±50.94 | | 18.55- 314.45 |
| SO_2_ (μg/m^3^) | 20.95±9.13 | 5.95-103.75 | | 21.51±14.02 | | 4.36-162.45 | | 21.04±14.27 | | 4.36-162.45 | | 20.26±15.10 | | 3.09- 176.74 |
| NO_2_ (μg/m^3^) | 50.72±11.57 | 13.71- 77.36 | | 50.46±16.17 | | 10.45-109.15 | | 50.38±16.30 | | 10.45-109.15 | | 51.47±16.63 | | 9.61-122.32 |
| CO (mg/m^3^) | 1.52±0.31 | 0.48-3.66 | | 1.56±0.56 | | 0.45-3.71 | | 1.52±0.56 | | 0.45-3.71 | | 1.49±0.58 | | 0.42-5.30 |
| O_3_ (μg/m^3^) | 50.57±11.99 | 7.37-88.30 | | 49.07±22.95 | | 7.13-106.15 | | 51.18±24.99 | | 5.02-112.34 | | 51.50±24.83 | | 4.64- 117.83 |

**Table S2** The effect of maternal exposure to air pollution during various periods of pregnancy on term birth weight, the risk of TLBW and macrosomia

| Exposure period | Air pollutant | Birth weight | | |  | TLBW | | |  | Macrosomia | | |
| --- | --- | --- | --- | --- | --- | --- | --- | --- | --- | --- | --- | --- |
|  |  | *β* | 95%*CI* | *P* |  | *OR* | 95%*CI* | *P* |  | *OR* | 95%*CI* | *P* |
| The first trimester | AQI | **-2.100** | **-2.576, -1.624** | <0.001 |  | **1.016** | **1.006, 1.027** | 0.002 |  | 0.986 | 0.982, 0.991 | <0.001 |
|  | PM_2.5_ | **-2.321** | **-2.819, -1.823** | <0.001 |  | **1.017** | **1.006, 1.028** | 0.002 |  | 0.986 | 0.981, 0.991 | <0.001 |
|  | PM_10_ | **-1.700** | **-2.088, -1.312** | <0.001 |  | **1.017** | **1.009, 1.026** | <0.001 |  | 0.991 | 0.987, 0.994 | <0.001 |
|  | SO_2_ | **-4.258** | **-5.408, -3.108** | <0.001 |  | **1.033** | **1.010, 1.057** | 0.006 |  | 0.974 | 0.963, 0.986 | <0.001 |
|  | NO_2_ | -0.798 | -1.720, 0.124 | 0.090 |  | 1.004 | 0.984, 1.025 | 0.695 |  | 0.979 | 0.970, 0.987 | <0.001 |
|  | CO | **-1.105** | **-1.394, -0.816** | <0.001 |  | **1.007** | **1.001, 1.014** | 0.025 |  | 0.993 | 0.990, 0.996 | <0.001 |
|  | O_3_ | **4.150** | **3.493, 4.807** | <0.001 |  | 0.963 | 0.949, 0.978 | <0.001 |  | **1.023** | **1.017, 1.030** | <0.001 |
| The second trimester | AQI | **-2.482** | **-2.925, -2.039** | <0.001 |  | **1.022** | **1.013, 1.032** | <0.001 |  | 0.987 | 0.983, 0.991 | <0.001 |
|  | PM_2.5_ | **-2.552** | **-3.054, -2.050** | <0.001 |  | **1.023** | **1.012, 1.034** | <0.001 |  | 0.987 | 0.982, 0.992 | <0.001 |
|  | PM_10_ | **-2.019** | **-2.359, -1.679** | <0.001 |  | **1.020** | **1.013, 1.027** | <0.001 |  | 0.990 | 0.987, 0.993 | <0.001 |
|  | SO_2_ | **-4.837** | **-5.895, -3.779** | <0.001 |  | **1.039** | **1.018, 1.061** | <0.001 |  | 0.975 | 0.964, 0.986 | <0.001 |
|  | NO_2_ | **-1.085** | **-1.984, -0.186** | 0.018 |  | 1.011 | 0.991, 1.031 | 0.287 |  | 0.983 | 0.974, 0.991 | <0.001 |
|  | CO | **-1.388** | **-1.674, -1.103** | <0.001 |  | **1.013** | **1.006, 1.019** | <0.001 |  | 0.993 | 0.990, 0.995 | <0.001 |
|  | O_3_ | **3.683** | **2.907, 4.459** | <0.001 |  | 0.968 | 0.951, 0.985 | <0.001 |  | **1.023** | **1.016, 1.031** | <0.001 |
| The third trimester | AQI | **2.274** | **1.865, 2.683** | <0.001 |  | 0.983 | 0.974, 0.992 | <0.001 |  | **1.013** | **1.009, 1.017** | <0.001 |
|  | PM_2.5_ | **2.344** | **1.914, 2.774** | <0.001 |  | 0.981 | 0.971, 0.990 | <0.001 |  | **1.013** | **1.009, 1.017** | <0.001 |
|  | PM_10_ | **1.705** | **1.363, 2.047** | <0.001 |  | 0.991 | 0.984, 0.999 | 0.023 |  | **1.010** | **1.007, 1.014** | <0.001 |
|  | SO_2_ | **3.270** | **2.237, 4.303** | <0.001 |  | 0.976 | 0.952, 0.999 | 0.038 |  | **1.016** | **1.007, 1.026** | 0.001 |
|  | NO_2_ | **5.219** | **4.331, 6.107** | <0.001 |  | 0.964 | 0.945, 0.984 | <0.001 |  | **1.021** | **1.012, 1.029** | <0.001 |
|  | CO | **0.855** | **0.580, 1.130** | <0.001 |  | 0.994 | 0.988, 1.000 | 0.070 |  | **1.005** | **1.002, 1.008** | <0.001 |
|  | O_3_ | **-3.258** | **-3.852, -2.664** | <0.001 |  | **1.024** | **1.011, 1.037** | <0.001 |  | 0.983 | 0.977, 0.988 | <0.001 |

*Note*: Above models were all adjusted for maternal age, gestational age and Ethnicity. Infants with normal term birth weight (2500g≤birth weight and <4000g, n=292192) were used as the control group while assessing the impact of air pollution exposure on the risk of TLBW and macrosomia.
